
JOURNAL INFORMATION
==============================
NLM Title Abbreviation: ScientificWorldJournal
Journal ID: ScientificWorldJournal
NLM Journal ID: 101131163
Journal ID: TSWJ

The Scientific World Journal

ISSN: 2356-6140
EISSN: 1537-744X
Publisher: Wiley

ARTICLE INFORMATION
==============================
PMCID: PMC6084530
PMID: 30147484
DOI: 10.1100/tsw.2001.141
Article ID: 821456
Article version: 1
Subjects: Short Report

Transepithelial Migration Process Induced by Interleukin-8 Delays Polymorphonuclear Leukocytes (PMNL) Apoptosis

Le'Negrate Gaëlle 1 *
Rostagno Phillippe 2
Rossi Bernard 1
Hofman Paul 1 3
1 INSERM U364, France
2 Laboratoire d'Oncogénétique, Centre Antoine Lacassagne, France
3 Laboratoire d'Anatomie-Pathologique, Faculté de Médecine, 06107 Nice Cedex 02, France
*Gaëlle Le'Negrate: lenegrat@unice.fr
Publication date: 2001
Electronic publication date: 2001 Oct 17
Volume: 1
First page: 38
Last page: 38
Copyright: Copyright © 2001 Phillippe Rostagno et al.
Copyright year: 2001
License: This is an open access article distributed under the Creative Commons Attribution License, which permits unrestricted use, distribution, and reproduction in any medium, provided the original work is properly cited.
License URL: https://creativecommons.org/licenses/by/3.0/

Funding: Institut National de la Santé et de la Recherche

==============================
Miami *Nature Biotechnology* Short Reports
*TheScientificWorld* (2001) 1(S3), 38SR
ISSN 1532-2246; DOI 10.1100/tsw.2001.141

### TRANSEPITHELIAL MIGRATION PROCESS INDUCED BY INTERLEUKIN-8 DELAYS POLYMORPHONUCLEAR LEUKOCYTES (PMNL) APOPTOSIS

Gaëlle Le'Negrate*(1), Phillippe Rostagno (2), Bernard Rossi (1), Paul Hofman (1,3)

(1) INSERM U364, (2) Laboratoire d'Oncogénétique, Centre Antoine Lacassagne, and (3) Laboratoire d'Anatomie-Pathologique, Faculté de Médecine, 06107 Nice Cedex 02, France
* lenegrat@unice.fr

**INTRODUCTION.** Acute colitis occurring in inflammatory bowel diseases (IBD) is characterized by massive migration of polymorphonuclear leukocytes (PMNL) across intestinal epithelium (1). This marked neutrophilic infiltrate was associated with enhanced mucosal interleukin-8 (IL-8) level (2). In such diseases, persistent acute inflammation delayed PMNL apoptosis, but the exact mechanisms underlying this phenomenon are still poorly defined (3). The aim of this study was i) to elucidate whether the transepithelial migration induced by IL-8 could influence the PMNL apoptotic process, and ii) to characterize the anti-apoptotic factor(s) involved in this event.

**METHODS.** Human epithelial intestinal "cryptic-like" cells (T84) were grown as inverted monolayers on 0.33 $cm^2$ ring supported transwell membrane and PMNL transmigration induced by IL-8 (100 nM) was examined. To reproduce massive PMNL transmigration, $5.10^6$ PMNL were added in the upper chamber and kinetics of PMN transmigration were carried out. Apoptotic status of transmigrated or non-transmigrated PMNL was analysed using flow cytometry for propidium iodide and TUNEL DNA staining, Annexin-V and $DiOC_6$ staining. Cell surface Fas and Fas-ligand expression was analysed by flow cytometry. Procaspases-8, -3, -6, and -7 expression was analysed by Western-blotting whereas " caspase-3 like " activity was assessed by caspase-3 assay.

**RESULTS.** TUNEL assay, Annexin-V and $DiOC_6$ staining revealed that PMNL apoptotic process was delayed in transmigrated compared to PMN exposed to IL-8, particularly between 6 h and 14 h of transmigration. Among potential anti-apoptotic factor(s), T84 cells by themselves or by the releasing a soluble anti-apoptotic factor are involved in this process.

Furthermore, although procaspases-3, -6, and -7 expression was decreased in transmigrated PMNL, caspase-3 activity remained weak in similar conditions. Transmigration was associated with a decrease in Fas-ligand cell surface expression on PMNL, although Fas remain functional in the same conditions. Moreover, inhibiting Fas-ligand cleavage by PMNL treatment with a matrix metalloproteinase inhibitor (BB94) induces an increased apoptotic rate in transmigrated PMN.

**DISCUSSION.** Taken together, theses data suggest that transepithelial migration process induced by interleukin-8 increases the life span of PMNL in the digestive lumen during intestinal inflammatory diseases. This process may increase host defence against bacteria located into the site of inflammation (4). Among anti-apoptotic(s) factor(s) potentially involved in this delay, our study demonstrates that interaction with T84 cells contributes to decrease PMNL apoptotic rate. We show evidences that Fas-ligand cleavage likely attenuates the homotypic Fas/Fas-ligand interaction in digestive lumen. Furthermore, decrease expression of procaspases-3, -6, -7 that occurs during the transmigration process might also participate in the delay of PMNL apoptosis program.

**ACKNOWLEDGEMENT.** This work was supported by Institut National de la Santé et de la Recherche.

**REFERENCES.**

1. Keshavarzian, A., Fusunyan, R.D., Jacyno, M., Winship, D., MacDermott, R.P., and Sanderson, I.R. (1999) Am. J. Gastroenterol. 94, 704-712
2. Brandt, E., Muller-Allouf, H., Desreumaux, P., Woerly, G., Colombel, J.F., and Capron, M. (1998) Eur. Cytokine Netw. 9, 647-635
3. Hennigan, S.M., Wang, J.H., Redmond, H.P., and Bouchier-Hayes, D. (1999) Shock 12, 32-38
4. Coxon, A., Tang, T., and Mayadas, T.N. (1999) J. Exp. Med. 190, 923-933
